# Veterinary student narratives of veterinary and interprofessional identity

**DOI:** 10.3389/fmed.2026.1748170

**Published:** 2026-02-17

**Authors:** Jessica L. Topka, Tamara S. Hancock

**Affiliations:** 1 College of Veterinary Medicine, University of Missouri, Columbia, MO, United States; 2 Department of Pathobiology and Integrative Biomedical Sciences, College of Veterinary Medicine, University of Missouri, Columbia, MO, United States

**Keywords:** dual identity, interprofessional collaboration, interprofessional education, one health, veterinary professional identity

## Abstract

**Introduction:**

The readiness for interprofessional collaboration (IPC) is rooted in interprofessional education (IPE). IPE shapes views of IPC and socializes students into professional roles. Previous work showed differences among medical, veterinary, and dual degree Master of Public Health (MPH) students in identity and role expectations using the Readiness for Interprofessional Learning Scale (RIPLS). Yet, it is uncertain which roles and identities are elicited while using this instrument. The authors sought to answer the following question: What professional and interprofessional identities do veterinary students construct through stories in response to RIPLS items focused on roles and identities?

**Methodology:**

Using think-aloud interviews with focused RIPLS items, 9 veterinary students shared narratives of veterinary and interprofessional identities related to constructs of the RIPLS. Within a social constructivist framework, data were analyzed using thematic analysis.

**Results:**

Analysis identified several complex narrated identities that overlap and inform the veterinary professional identity, including veterinarian, veterinary interprofessional, interprofessional, and One Health identity. Participants narrated veterinarians in stories as communicators with a wide range of professional roles who must advocate for themselves and the status of their profession. Veterinary interprofessionals were multi-level team players within a veterinary healthcare team that collaborate to achieve common goals, with an emphasis on maintaining team safety. Interprofessional identity encapsulates skills and attitudes that veterinary medicine shares with other healthcare fields, yet employs them independently, which were discovered by participants through casual interactions with people trained in other fields. Participants discussed One Health as an important interprofessional space where veterinarians collaborate with other fields for a ‘greater good’. Ambiguity regarding identity and roles emerged in relation to the RIPLS.

**Discussion:**

These findings affirm research that situates veterinary professional identities as complex, contradictory, and dual. One Health orientations resonate strongly with the veterinary community and may be better suited for IPC inclusive of veterinary medicine. Social media and social interactions have potentially significant impacts on veterinary professional identity development and remain underexplored. Participant confusion surrounding RIPLS items contributes to criticisms of the tool, widely discussed in IPE/C literature. Further work is needed to identify the best tools and approaches to investigate veterinary professional identity and attitudes toward IPC.

## 1 Introduction

Contemporary public health challenges require One Health approaches that mobilize multiple professional communities into a unified effort. One Health is defined as “an integrated approach that aims to sustainably balance and optimize the health of people, animals, and ecosystems” (1). Public health, inclusive of One Health, has become integral to many curricula of professional programs of veterinary medicine (PPVM) as a dedicated day one competency (2), and recent work indicates all veterinary students have access to One Health curricula (3). One Health positions veterinary professionals as collaborators at the nexus of human, animal, and environmental health.

Interprofessional collaboration (IPC) is defined as “multiple health workers from different professional backgrounds work-[ing] together with patients, families, carers (caregivers), and communities to deliver the highest quality of care” [(4), p. 8]. Interprofessional Education (IPE) “occurs when students from two or more professions learn about, from, and with each other to enable effective collaboration and improve health outcomes” (4). IPE enables IPC, and IPC is vital to tackling contemporary health issues, e.g., the COVID-19 pandemic (5) and antimicrobial resistance (6).

Considering their definitions, individual instances of IPE and IPC may fall under the auspices of One Health, but not all IPE and IPC experiences fully embody the underlying key principles of One Health. However, One Health and IPE are also frameworks consisting of competencies used by academic institutions to teach collaborative practice (3). IPE frameworks are commonly employed at medical institutions, while One Health frameworks are more common in veterinary programs (3).

Previous work illustrates how the development of a shared goal promotes IPC (7) and required IPE experiences confer benefits to veterinary students in exchanging ideas on common practices, e.g., compounding pharmaceuticals (8). Veterinary students describe their roles as educating other professions and the public about the necessity of veterinary medical expertise and how it benefits society (8, 9). In contrast, only approximately 60% of medical students have access to One Health curricula (3), and many medical students lack knowledge about the roles and competencies of veterinarians (9). Successful IPC must acknowledge and navigate the tensions of these closely linked and inter-dependent health systems. Previous work illuminated differences among professional student groups [i.e., Doctor of Veterinary Medicine (DVM) vs. Doctor of Medicine (MD)] at a single institution and showed that differences in identity, group work, and role expectations among medical, veterinary, and MPH students are key factors in IPE and thus IPC (10–12). Yet, more work is necessary to explore what identities and roles are operationalized.

Veterinary medicine is largely omitted from reviews exploring health professionals’ readiness for IPE and IPC (13) or longitudinal projects following IPE and IPC in various healthcare fields (14). Rather, IPE and IPC in veterinary medicine often fall under One Health, which is operationalized as “a collaborative, multisectoral, and transdisciplinary approach—working at the local, regional, national, and global levels—with the goal of achieving optimal health outcomes recognizing the interconnection between people, animals, plants, and their shared environment” (15). Roopnarine and Boeren (10) indicated that DVM students are more ready for IPE and IPC, more teamwork oriented, but less secure in their professional roles, in contrast to MD students. Furthermore, MD and DVM students who concurrently pursue an MPH degree have a more robust understanding of One Health (12).

Roopnarine and Boeren (10) utilized the Readiness for Interprofessional Learning Scale (RIPLS) to identify these differences and started to fill the gap where veterinary medicine is missing from IPC research. The RIPLS is a commonly used tool to investigate readiness for IPE/C. It has been translated into several languages and has assessed student populations across the globe (16–19). In response to its widespread use, several criticisms of the RIPLS have emerged. In the multiple translations and versions of the RIPLS, different items and constructs have been identified as showing misfit and low validity, respectively (16, 20–22). Findings of unstable reliability and factor structures have resulted in conclusions that the RIPLS should be used with caution and refined with further work (18–22).

The success of IPE and IPC can be impacted by facets of professional identity. Professional identity is a socialized, in-group identification with one’s professional group (23–25). The construct is often tacit yet can be more actively targeted for development (26). An individual’s personal and professional identities are expressed in varying strengths based on context, hierarchy, and emotional state, among other competing factors (25).

Traditional health professions education acts to socialize students into their own profession and professional identity. This produces members highly committed to their profession and have internalized a strong sense of belonging to their profession or cohort (27). Although socialization has many benefits, it can present barriers to collaboration through loyalty and a lack of knowledge about interprofessional colleagues (28). Indeed, professional identity formation can create professional silos that can undermine the potential for IPC within educational settings (29).

The development of a dual identity is proposed as a means to leverage the strength and expertise of multiple professions (27, 30). Importantly, dual identity involves robust belonging and orientations towards both one’s own profession and the IPC community one is also a member of (24). This identity can be developed through shared work (31) and can be successfully targeted in educational interventions when students are becoming socialized members of their profession (24). Sims (32) found that dual-trained professionals had more flexible professional identities and were comfortable with ambiguous or unfixed duties, which may also extend to positive impacts in IPE and IPC. Additionally, the establishment of a common goal through One Health is suggested to facilitate IPC across human and veterinary healthcare (7).

Narratives provide a robust mechanism for interprofessional identity construction and investigation. Previous work reveals a multiplicity of identities constructed by clinicians and students within interprofessional interactions (33). Furthermore, these identities are constructed through narratives to make sense of identity development embedded and imbued with social norms. Role models are central characters in student IPC identity narratives, which emphasize the power of social figures of status in shaping the development of interprofessional identities (28, 33).

Much is left unexplored across readiness for IPE across veterinary programs. Research is needed to expand our understanding of IPE/C orientations in a veterinary context so that IPC is better facilitated and strategically approached to mitigate our next global health challenge. Work to describe veterinary professional identity is still emerging, with scant work elaborating how veterinary students position themselves as having dual identities. Furthermore, clarity on the discourse surrounding IPE and IPC is needed. It is uncertain if veterinary healthcare professionals are considered collaborators in an interprofessional setting when IPC or IPE discourses are examined.

Based on the need for unified One Health and IPC efforts that reach across the globe, a deeper understanding of differences among veterinary professionals and across IPC groups is necessary. Considering these gaps in our understanding, we sought to examine the following research question:

1. What professional and interprofessional identities do veterinary students construct through stories in response to RIPLS items focused on roles and identities?

This study begins to fill the complex gap in understanding IPE/C orientations and identities among veterinary professionals. Insights from this study can inform the need for expanded IPE within veterinary curricula, support the development of dual identities or other collaborative competencies to support IPC, and support the success of such programs. Finally, these findings will add to the work that builds capacity for the inclusion of veterinary professionals in IPE/C experiences as we work together to improve global health.

## 2 Materials and methodology

### 2.1 Ethics

The University of Missouri’s institutional review board approved the project and provided it an exempt status (MU IRB Project #2090254). All data production and participant communications were via one researcher with a peer status (fellow professional degree-seeking student) at the time of the research (JLT) to minimize influences of power and status on participants seeking to participate.

### 2.2 Theoretical framework

We used a social constructivist framework (34). This theoretical approach situates the construction of the self via discourse and practice within a social and cultural context. Communication conveys messages and makes claims about who the speaker is relative to other figures in that context and the nature of the relationship to those figures. Neither social nor cultural discourses and practices are static nor universal, but rather improvisations of a self within a sociocultural practice, rather than a self in essence. Identity develops at this interface, where individuals self-author their identities through selective curation of perceived norms, values, and other figures within the worlds they associate with or wish to associate with.

Narratives are recounting of stories told by participants that connect events and construct identities they see as relevant to IPC (35). Narrative identities are fluid improvisations of one’s sense of self produced through the communication of the complex interfaces surrounding the self, social belonging, status/power, and a future self (identity-to-come) that may be called upon to engage in IPC.

### 2.3 Participants

Interview participants consisted of nine DVM students, three of whom were DVM-MPH dual degree-seeking students. They were in one of the first 3 years of study in the same PPVM, which was selected for convenience. The PPVM has a required didactic Public Health course that delivers content related to the veterinary aspects of certain diseases or public health issues without consideration for collaboration. Otherwise, there are no formal or elective IPE training at this PPVM. Eight of the participants are women, and all espoused a variety of interests in the field of veterinary medicine, reflecting contemporary demographics of the profession. All participants were assigned a pseudonym for anonymity. Further participant details are summarized in Table 1.

**Table 1:** Summary of participants.

**Pseudonym**	**Year of Study**	**Degree program(s)**	**Field(s) of interest**
Amy	VM1	DVM	USDA, regulatory medicine, agriculture
Bethany	VM1	DVM, MPH	Wildlife conservation, zoo and exotics medicine
Michelle	VM2	DVM	Small animal general practice, One Health
Camile	VM2	DVM	Undecided, research, surgery
Mason	VM2	DVM	Undecided, small animal general practice, One Health
Kaitlyn	VM2	DVM	Undecided
Taylor	VM3	DVM, MPH	Laboratory animal medicine
Morgan	VM3	DVM	Laboratory animal medicine
Ashley	VM3	DVM, MPH	Small animal general practice, laboratory animal medicine, possibly PhD later

### 2.4 Data production

Participation in the study was entirely voluntary. Consent for participant recruitment was obtained from the institution where recruitment occurred. Recruitment occurred through emails distributed to DVM students via institutional distribution lists. These emails were approved by the IRB and listed the researchers as contacts, but were not sent from or signed off on by either researcher. These emails described the project and aims, explicitly indicating that the research intended to explore the roles that veterinary medical students take up when they engage in interprofessional learning and in what ways students are ready for interprofessional work. Approved informed consent documents were attached to the email. Prospective participants indicated their interest in participation via a hyperlink to a survey that collected their email addresses. Prospective participants were contacted via email, and a mutually agreeable time was arranged between one researcher (JLT) and the participant.

Think-aloud protocols (TAP) (36, 37) are interviewing techniques that can provide insight into thought processes and are commonly used to improve survey items. TAP techniques were used to inform the semi-structured protocol, as these techniques are demonstrated to better understand the deliberations around these items and thus the negotiation of identity with regard to IPC. With the limitations of the RIPLS instrument in mind, we elected to utilize RIPLS to further investigate veterinary student attitudes toward IPE/C. The RIPLS is an instrument consisting of nineteen to twenty-three 5-point Likert scale items (38, 39). It is a four-factor instrument that measures teamwork and collaboration, negative professional identity, positive professional identity, and roles and responsibilities.

Interview protocols were semi-structured and developed as a think-aloud conversation focused on RIPLS items 10–19 (39). These items were selected as they focus on constructs salient to professional identities, i.e., Negative Professional Identity, Positive Professional Identity, and Roles and Responsibilities. The semi-structured protocol was piloted with three veterinary faculty and revised to improve the flow and conversational nature of the interviews. Interviews lasted between 30 and 60 min and were audio-recorded. Interview transcripts were initially transcribed verbatim utilizing Otter.ai (40). All generated transcripts were then reviewed and revised as needed for accuracy by one researcher (JT) to protect participant anonymity.

### 2.5 Data analysis

Narrative analysis of transcript data was performed to examine what interprofessional identities were discursively produced and how they were constructed. First, both researchers engaged in a primary reading of transcripts with open coding to identify salient stories of roles and identity to focus and organize data. These codes included skill sets such as communication and collaboration, professional identities such as healthcare professional or veterinarian, and roles such as team members. These coded stories described influential events and articulated the participants’ role and/or relationship to other figures during the event. During these initial impressions, jottings and analytical memos were generated separately by both researchers to capture impressions of constructed identities and then discussed collaboratively to refine our organization of data and direct our next analytical steps. Next, we engaged in a close reading of coded excerpts to generate descriptions of what narrative identities were produced. Then we independently aggregated and coded data into identity groups salient to the research question (e.g., veterinarian and interprofessional). We drew on our theoretical framework (26) for a deeper analysis of how storied identities were produced in the moment with regard to various figures in their figured worlds. These deeper details include the status of identity (e.g., relative to other professions or figures) through indexing (e.g., us/them/we), those roles operationalized within identities (the spaces people occupy, skills they leverage, and why), and the nature of collaboration (their relationship with that space including emotion, dis/comfort, experiential, hypothetical, etc.). These details were collapsed into master narratives for these identities. Finally, we mapped out salient dimensions of these master narratives, including figures, relationships, and status, and generated four distinct yet overlapping professional identities.

### 2.6 Reflexivity

This study is situated within a paradigm that acknowledges the co-construction of data between interviewer and interviewees and thus necessarily involves reflexive practices. Because one researcher (TSH) was a faculty member who therefore occupied a position of power, the researcher who was considered a peer (JLT) performed all interviews. Both members of the research team maintained reflexive journals where tensions, preconceptions, and other reflections were recorded, then discussed and examined further in weekly and biweekly check-ins.

## 3 Results

### 3.1 Narrated identities

**Figure 1:**
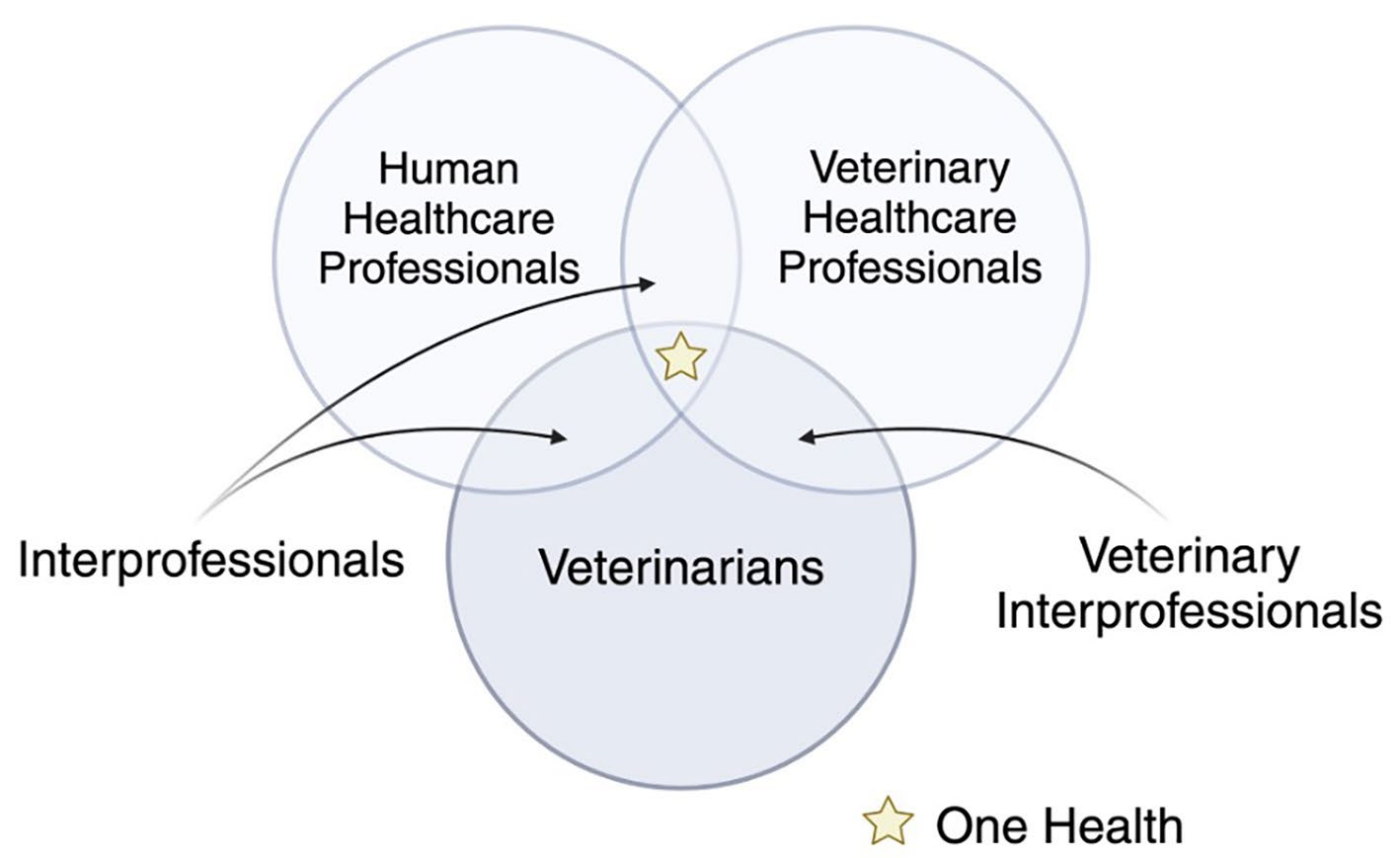
The interrelationships among participants’ narrated identities.

Stories of collaboration surrounded the professional identities of veterinarians, interprofessional, and One Health. Veterinarian identity is shared connections that produce the veterinary profession as a cohesive unit and mark participants as belonging. Veterinary Interprofessional identity is produced within a veterinary healthcare setting and is focused through a shared goal of patient (health)care and maintaining the safety and wellbeing of the entire veterinary healthcare team. The veterinary interprofessional encompasses collaboration within the wider veterinary community (i.e., other veterinarians, veterinary technicians, assistants, receptionists, office managers, and other colleagues). Interprofessional identities are those that are skills and tasks shared across health professions (e.g., both veterinarians and physicians have to deliver difficult news). Many of these stories arose from personal experiences (e.g., patient or caregiver) or familiar relationships with someone within the human healthcare sector (e.g., friend and family member). Some of the sharpest demarcations between the identities of veterinarian and interprofessional were the contrasts that excluded participants from interprofessional identities (e.g., where skill sets differ). One Health identities are stories where participants visualize themselves as having desirable competencies within a shared One Health scenario where human, animal, and environmental health are intertwined. Most participants narrated an unquestioned assumption that IPC with veterinarians and human healthcare exists primarily under the guise of One Health. The interrelationships among these narrated identities are represented in Figure 1. Each of these are explicated in more detail through the voices and stories of our participants below. Identity and Role Confusion emerged in our analysis as participants grappled with the instrument. They posed questions and struggled to orient themselves and their identities in relation to certain constructs. We have represented participants’ verbatim quotes in meaningful ways that preserve the tentative nature of their narrated identities.

#### 3.1.1 Veterinarian

Participants often narrated contradictory spaces and dualities of veterinary identity. More extreme dualisms are drawn from social media posts, memes, or other ‘folk lore’ of the veterinary community that reifies perceptions of lower status versus physicians or human healthcare. Some participants internalize this as truth in their story, while others situate themselves elsewhere.

*[And] I think that there’s probably been a lot of pushback in our, and within our, community in order to be like, recognized, you know… we are medical students, or we, you know, we’re not human medical students, but we study medicine* (Camile).

*I guess … that comes off a little bit conceited to me … the emotion that evokes for me is somebody who would say that their position is more intensive than any other health care physician … I don’t really think there’s too many health care, like students that have to learn so much more than other health care students* (Bethany).

Participants draw on conversations with non-veterinarians and experiences with medical students, family members, and members of the public to shape a professional identity that has veterinarians as hard-working and deserving of higher status than is often offered. Participants shared this in a way that at times seemed personal, and they reacted defensively. Other times, the ‘more than one species’ was embraced as a positive aspect and not a social detriment.

*Vet students, a lot of times, you get both ends of the spectrum of people being like, ‘Oh, you're **just** going to vet school,’ versus ‘Oh, you're going to vet school, you're better than those med students because you have to learn about 14 species.’ So you have to find a way to balance both of those extremes. And I think that just depends where you'll fall on that spectrum is just a very individual and personal thing that does, I think, depend on who you interact with, whether that's people in your discipline versus outside of your discipline, because you have to have that respect for those other professionals because you have to work as a team* (Morgan).

There was a diverse and often fragmented range of professional competencies afforded to veterinarians in participants’ stories. Bethany articulates the point with regard to educational experiences and how this is confounding, “so it’s kind of just like, they try to throw [collaboration] in randomly, and it just doesn’t really work because it’s just kind of like a small part of a bigger, just kind of solo journey” (Bethany). Other participants describe interactions that see the diversity of trajectories as a positive.

*I've heard it from a lot of other vets … that's like, oh, yeah, I used to work with pigs. But now I'm working with horses or I used to be a small animal practitioner, and then I moved into, you know, lab animal medicine. Some people work in different government roles, but they started in private practice. Like, there's so many different ways to work in a lot of different areas. … I've seen other people take their veterinary degree and do a lot of different things with it.* (Ashley)

Participants located veterinarians in stories as communicators, interconnected at the nexus of humans and animals across many disciplines (e.g., nursing, human healthcare, and public health). They must advocate for themselves and the status of their profession due to public misperceptions of the roles of, training for, and professional nature of veterinary medicine and veterinarians.

Veterinarians have a wide breadth of knowledge to assume responsibility for the vast array of species they work with. The breadth of knowledge allows veterinarians to practice in many suboptimal spaces, e.g., fashioning ‘fixes’ out of ordinary materials and sharing these remedies in social media, creating spaces that serve as markers of status in collective knowing and being on social media. In some ways, these points of pride conflict with the status for the veterinary identity they desire (i.e., on par with physicians) and the status they perceive (i.e., not recognized as “real doctors”). Many participants provided discursive positioning that regards veterinary medicine as misunderstood and unacknowledged for the expertise, time, and effort of training, which they supported with what they have heard or seen, often on social media.

*But this like, thing that, that [veterinarians are] not, we're not real doctors, right? Like, yes, we go to school for it and everything. But a lot of people when I was applying and stuff, were, like, would ask me questions about the school, they'd be like, oh, so like, is that like, something you do? Like, is that like, like, a two-year program? Or like, or Oh, is that just like a, you know, an undergraduate degree? Like, there's definitely not a lot of knowledge about what's involved in becoming a veterinarian* (Camile).

*I guess in general, like a lot of people don't know about the veterinary discipline or what that really involves, like, I mean, I've had people even ask me, like, Oh, that's a two-year degree program, right? And I'm like, No, I'm being a doctor that usually is four years, at least in most doctoral programs* (Bethany).

Participants’ stories suggested that the public should already know the kinds of training and education that are involved in becoming a veterinarian, and the fact that people were unaware served to reify a lower status.

#### 3.1.2 Veterinary interprofessional

The veterinary interprofessional is a multi-level team player within a veterinary healthcare team that collaborates through communication, shared learning, mutual respect, and listening to achieve shared goals of patient healthcare, public health, and maintaining the safety and wellbeing of the entire team. The latter of which was articulated as especially true for helping protect and support technicians’ (nurses) social and psychological safety. Our participants primarily described the veterinary interprofessional team within a veterinary clinic or hospital setting. The figures that participants drew upon were those veterinarians they previously worked with as former assistants/technicians/nurses.

A major facet of the veterinary interprofessional team was maintenance and support for the safety and wellbeing of the entire healthcare team. This role places veterinarians in a different register of power in the ways that they must both share power and knowledge and then take on power in a supervisory role for the safety of the same group of coworkers. Taylor was direct, “you also have to recognize that we also have vet techs that we have to look out for … and make sure that they’re safe and protected.” This stemmed from an experience prior to veterinary school.

*We had a client come in with a big English bulldog … for a blood draw. So, it was just us techs working and we went in for the blood draw and were having a hard time and the client was very … [she] already just came in with a bad mood, and we were a younger team of vet techs. So, she just was kind of untrusting of us. So, I went back to talk to the doctor, because [the client] had demanded that she had made an appointment with the doctor… But we explained that to her very nicely, okay, we can go back and see if the doctor is available. The doctor was on his way out the door and said, Sorry, you guys can deal with it, like, you guys can try to figure it out. I'm going home. We're like, okay, and then it ended up escalating to the point where we had to call the police because she was getting belligerent with us. Having that sort of support there would have been much appreciated* (Taylor).

Several participants shared similar stories of feeling unsupported when they were in unsafe situations at their veterinary workplace. Such as Ashley, who indicated, “I have a lot of memories from working before starting that school, where there was not necessarily the best work environment.” These experiences are shared and reified on social media, which further influences participants’ conceptions of a veterinary interprofessional team.

*…Because I've seen working in clinics and also just being on social media and seeing these discussions in different groups, like, it's really difficult when support staff are in a clinic environment where they don't feel supported by the veterinarian, or the clinic owner, or a manager* (Amy).

These experiences shape their investment in staff safety and respect as a common goal that defines and actions that produce the veterinary interprofessional.

*Just in general, as working as a veterinary technician, or veterinary assistant, I found my work was much more conducive with veterinarians that respected me as a worker, instead of just treating me as like a, like a foot servant or something. I just don't find it was better for either me or the patients … personally, I would never want to work with a veterinarian that doesn't respect their technicians, no matter how talented they are as a doctor* (Bethany).

Common goals were articulated as a central piece to narratives that focused on the veterinarian as an interprofessional embedded in a clinic and community. Camile articulated this in stories about teams, mentioning “everybody should be providing support for each other,” and that importantly, this was a mutual respect of different skill sets and expertise that “should be working towards that common goal.” This common goal was patient healthcare or public health. Public health was emphasized through the roles of veterinarians in communities and as stewards. Skill sets often articulated as shared and supportive of common goals were learning, respect, listening, collaboration, and communication.

#### 3.1.3 Interprofessional

Interprofessional identity encompasses those competencies that veterinary medicine shares with other healthcare fields, yet employs them independently in silos. For example, a veterinarian and a veterinary technician team working in a clinic parallels a physician and a nurse team working in a clinic. These shared skills include problem solving, communication, delivering difficult news, medications, clinical reasoning, and procedures and tools. These are typically learned or performed independently yet discovered as mutual through casual conversations, personal experiences, and social media. Participants emphasized how recognition of this interprofessional identity has the power to improve patient outcomes and enhance respect for veterinary medicine as a profession. Kaitlyn articulates this impact, “So there might be less misunderstanding to help communication.” This was important as many viewed the veterinary identity status as less than those of physicians and the hope was pinned to mutual realizations that many facets of practice are common across healthcare disciplines. Ashley indicates “clinical problem-solving skills can be learned… in both professions. And I don’t think it would be bad [for physicians] to get some different perspective on you know, [veterinarians] use XYZ diagnostics and look for these signs like… I think it’d be very applicable to both fields.”

Participants narrated themselves outside of other disciplines and healthcare fields in these stories, despite sharing so much. For some, it was positioned as a benefit, and for others, it was another source of distance that contributes to misconceptions and a lack of status. Amy shared several stories of casual conversations that informed this identity, for instance:

*My neighbor is a pediatrician. … We have conversations sometimes, like not telling each other how to communicate, but just sharing experiences, and I find he also has noncompliant patients, right? … we all certainly have noncompliant patients in vet med, like clients, you know. And so just listening to those like realizing, yeah, a lot of what you deal with is the same thing I deal with just like a little bit different.*

Michelle shared stories of going home during holiday breaks and reconnecting with friends who were in medical school where they discovered and were shocked by this shared, yet separate interprofessional identity. “We were having these casual conversations, and there were so many parallels between both that were just shocking … I think we’re all kind of in our own little professional worlds.”

In other ways, friends or family in another medical field influences participants’ perceptions of how they are similar yet separate from human healthcare. Mason’s sister and many friends are in medical school, and their mom is also a certified nursing assistant. Amy’s mom is a nurse. Camile drew on caregiving experiences to inform this facet of her identity, “my perceptions on this have definitely been guided by my mom’s healthcare, concerns in the past and the way that she has interacted with her doctors.” Despite these similarities, there was also a sharp demarcation that separated veterinarians.

*I think that like a lot of our schooling is very, very similar and that the way that we approach a lot of these things are very similar. There are some important differences. And not, which is not to say that one is better or worse than the other. I've just noticed some differences in the way that each thing, each path sort of looks at things as a whole is like a, like a broader perspective. I… I think, in the way that vets tend to communicate with or see a clinical issue and then the way that doctors are used to obtaining that information* (Camile).

*With other healthcare students, some not all outside of the veterinary spectrum, just because things aren't taught the same … like, you know, Salmonella. If I'm learning about Salmonella and pigs, and they want to talk about Salmonella in people, you might not be [focused on] the same thing… in undergrad, all my friends were pre-med and so like, when I would talk about things, they would try to correct me. It's completely different* (Morgan).

This act of “correcting” Morgan reinforced a difference in status between veterinarians and physicians.

#### 3.1.4 One health

One Health was essential to the veterinarian identity, and this exists in an important interprofessional space where veterinarians can cross disciplinary boundaries and collaborate with human healthcare and other sectors for a ‘greater good’. Participants described awareness through coursework, clubs, or mentorship in addition to experiences they have had that related to a One Health approach to outbreak management, global health, and psychological wellbeing, to name a few.

Amy told stories from their undergraduate mentor that informed One Health identity as a collaborator constrained by their own disciplinary boundary.

*I've never been on like a zoonotic disease outbreak. But my mentor went to an Amish herd one time, a dairy herd, where like 70% of the cattle tested positive for TB, and the kids in that community were drinking the milk. And she was talking about how as a DVM, she's not allowed to make human health recommendations. So she tested the cattle, right, she decided what to do with them. But at that point her—the jurisdiction basically stopped. And then she had to call the health department and then wait for them to come out, and then advise those people on what to do. And even though she technically had the knowledge and wanted to be like, stop drinking the milk, and you need to rush on your kids to a doctor, and, you know, this might be really serious. And so sort of, we both have to stay in our lane. But at least if we can understand the other, and maybe even just have connections to resources, like to better funnel patients off either way, is helpful.*

Michelle has been involved in research with animal models of disease and has seen the positive impact of these collaborations, which, in turn, has made them “most passionate about this collaboration between vet med and human med.” Many participants discussed how siloed healthcare fields are and how One Health is or should be a space for vital collaborations. Bethany went so far as to say that “our professions should be interrelated because in the greater world, like, we’re working on similar things, and if we work together, we might actually be better at solving them.”

Some participants articulated how One Health orientations across different professional disciplines may serve to improve communications among these groups that ought to be working together. “Ultimately, I think that One Health is really important … I think it helps me appreciate where they’re coming from, and maybe sometimes recognize when, you know, there does seem to be not hostility, but like misunderstanding, I guess, between different professions” (Taylor).

#### 3.1.5 Identity and role confusion

Throughout the analysis, we found that eight out of the nine participants expressed confusion regarding the RIPLS instrument. They thought aloud quizzically and desired clarity so they could locate their identities with the constructs presented. In particular, items within the positive professional identity and roles and responsibilities were common sources of uncertainty. For instance, the nature of patient problems (positive professional identity construct) was a frequent snag:

“[I’m] not really sure about the word nature of patient problems in this case, but… yeah, this one’s kind of stumping me” (Michelle).

“I'm not sure. Sorry, trying to think through it… when you say patient problems, what is the implication?” (Camile).

“So I'm assuming shared learning also, is shared learning with other healthcare professionals or other healthcare students, right” (Taylor)?

“It says other than other healthcare students, which doesn't necessarily mean other doctors, well, that is supposed to mean just like MD students” (Amy).

“What’s the, like, nature is like the etiologic can’t even pronounce the thing, you know, what’s actually causing the problem? Yeah. Or is it like, something else that… like the environment? As like, I don’t really know how to go, or like, answer this statement as much” (Mason).

“I think I don't understand the meaning of clarify the nature of patient problems, like I don't have a concrete idea of what that would look like” (Ashley).

“I don't really understand then. What do you mean by nature” (Kaitlyn).

The roles and responsibilities items produced puzzlement. The direct question regarding professional roles created a roadblock for participants, unable to locate themselves or any stories regarding veterinary identities. Kaitlyn responded with a question, “I’m not sure what my professional role will be… Will be in what?” Mason similarly wondered what was implied and posed additional questions, “Is it asking like what I want to do in the future? Like, what my job or like what I’m supposed to do?” Bethany implied the item read duplicitously,

*I know my professional role will be with that, if as long as I graduated, obviously, will be that of a veterinarian. I guess. I'm not sure. That's a weird question. Man, that is weird. But like, just, I guess it just doesn't seem like it doesn't provide a specific like, it just seems kind of weird that you would graduate and not know what your professional role would be?*

Participants also dissected the discourse in another item in this construct, regarding the roles of nurses and therapists. “So it says the *function* of nurses and therapists—I don’t know. I feel that it feels like they’re like tools. Because they feel the need to say function. Yeah. Oh, it may be better to say role or something” (Kaitlyn). Bethany explicates,

*I just don't necessarily like how the question is worded. The function of nurses and therapists is mainly to provide it just, it's sort of just worded in a way that they just don't find tasteful, as if like, that's their only function or like, that's the only reason they're there is just because of the doctors is, like that whole idea, I guess that the doctors are at the pinnacle, and everybody else is like below them. I don't like that idea.*

She goes on to describe how the language of the item itself shifts her answer, “So I guess in that case, I’m going to have to answer neutral because I just don’t prefer how the question is worded” (Bethany).

## 4 Discussion

Scholarly understanding of IPE/C identities in a veterinary context is necessary so that IPC is fostered and strategically approached to mitigate global health challenges. The literature surrounding veterinary professional identity is emerging, yet it lacks a robust characterization of IPE, IPC, or dual identity aspects. This study describes veterinary professional identity through narratives that capture veterinary student orientations around IPE/IPC prior to graduation.

Veterinary students responding to RIPLS items frequently shared narratives describing veterinarians, veterinary interprofessionals, interprofessionals, and participants in One Health initiatives as identities-to-come. These momentary improvisations reflect their negotiation and navigation of existing figures and identities communicated via educational programs, peers, mentors, and social media as they are themselves becoming these identities. Longitudinal work is needed to elucidate the trajectories of professional identity development, inclusive of pre-veterinary and post-graduate veterinary work, to characterize the continuum of identity from (pre-)veterinary student to veterinarian.

Veterinarian identities are multifaceted and contradictory, affirming other accounts of narrative veterinary identities (41). Stories of veterinary professionals working in diverse environments (e.g., hospitals, farms, government offices, laboratories) with a wide range of species as patients were shared, and some described this role confusion as a barrier to determining their veterinary identity. Such a barrier may explain Roopnarine and Boeren’s (10) conclusion that DVM students were less secure in their professional roles in contrast to MD students. Emphasis on status, or lack thereof, was a consistent theme in participant narratives, which drew on stories exemplifying non-veterinarians’ lack of knowledge surrounding the duration and composition of veterinary medical education. Englar et al. (9) confirmed a lack of knowledge in human healthcare students regarding the length of PPVMs, availability, and breadth of specialty options in veterinary medicine, and veterinary roles beyond that of the practitioner (e.g., public health and environmental conservation). However, it is unclear if this described lack of knowledge can be used as direct evidence that veterinarians are not recognized as “real doctors” or are universally deemed lower status compared to physicians. Englar et al. (9) express interest in “[exploring] how *veterinary* students perceive *human* healthcare professions”, which may elucidate bias and lack of knowledge veterinary students have toward health professions of supposed higher status. More work is needed in longitudinal explorations of interprofessional and dual identities that incorporate veterinarians. Recent work further emphasizes the importance of early exposure to IPE before graduation and practice in a variety of healthcare sectors, but excludes veterinarians (14), perhaps because of the focus on IPE rather than One Health.

Participants discussed the role of a veterinary interprofessional in supporting the entire veterinary team and protecting their psychological safety. Drawing on previous experiences, they constructed an identity encompassing the responsibilities of a veterinarian not only to their patients, but to their coworkers. Interestingly, the skill sets of a veterinary interprofessional (e.g., learning, respect, listening, collaboration, and communication) described by participants strongly align with Gittell’s theory of relational coordination (RC) (42). Blokland et al. (43) summarize, “RC describes a mutually reinforcing process of communicating and relating… and relating is characterized by shared knowledge, shared goals, and mutual respect.” Literature investigating RC in human healthcare teams has linked stronger RC scores to increased psychological safety, increased job satisfaction, increased work engagement, reciprocal learning, decreased burnout, and decreased turnover (43). One study examining 578 employees of veterinary hospitals found that higher RC was associated with a healthier perception of psychological climate and increased job satisfaction (43). Participant descriptions of veterinary interprofessional identity may suggest a potential intervention regarding RC and strengthen the future veterinary teams that students will join throughout their careers. Additionally, RC may be a useful tool to evaluate teams in IPE and IPC settings.

Veterinary students articulated dual identities of veterinarian and veterinary interprofessional, like physicians (27, 28, 30). Both DVM and dual degree DVM-MPH participants produced narratives highlighting veterinary roles consistent with dual identity. Dual identity situates veterinary professionals as responsible for their own professional work in addition to a responsibility toward a greater good, which in this context is a shared goal of physicians and other healthcare professionals. Importantly, dual identity involves both a robust belonging toward one’s own profession and the IPC community (24). Sharing stories from the workplace, participants emphasized the importance of veterinary medicine as a team effort embedded within a clinic or community. Common goals include patient healthcare and outcomes, as well as extending beyond veterinary patients to public health and community wellbeing. Many stories connected these orientations to other healthcare fields, expanding discussions to the IPC community. Participants identified parallels between veterinary and human healthcare when speaking with neighbors, friends, and family members within the field. These stories are key for interprofessional socialization, which facilitates better IPC (24). Conversely, Cantaert et al. (44) argue that dual identity detracts from group distinctiveness and blurs role boundaries. Think-aloud interviews with veterinary students revealed a context that positively situates veterinary professionals to develop dual identity, and more work is needed to characterize the development of dual identity throughout veterinary education and determine how it benefits or detracts from IPC initiatives.

One Health is a comfortable term and a frequently referenced context for veterinary student participants considering IPC. This finding affirms those of Tucker et al. (3) among veterinary medical and human healthcare educators, which indicated that One Health competencies resonated with veterinarians more than IPE competencies. Both frameworks are similar in theory but diverge in practice because terms belong to siloed groups. Veterinary student participants narrated interprofessional identity as a means of connecting to other professions through shared yet separately utilized skills, while One Health was discussed as shared approaches or actions taken to achieve common goals. This seemingly contrasts with interprofessional/interprofessional education terminology for medical professionals, and more work is needed to better characterize how physicians and physician trainees conceptualize and name these spaces. Additionally, more work is needed to investigate how different frameworks and terminology across professions can be leveraged to better facilitate IPC.

One Health identities in our data captured stories of veterinary collaboration across healthcare sectors. Participants expressed comfort framing collaborative work in the context of One Health. Importantly, they emphasized that One Health work not only drives progress in public health sectors but also fosters understanding among professions, which bolsters the suggestion for One Health as a “clear common goal” to facilitate IPC (7). Although the jurisdiction of different professions was also considered frustrating in scenarios when time is of the essence, these responsibilities simultaneously enforce the importance of diverse expertise. One Health may be a more attainable gap to close across the professions compared with IPE, as recent work characterized all veterinary students and 60% of medical students having access to One Health curricula (3). One Health identity may be an effective focus for the veterinary profession; however, more work is needed to determine if it is an effective lens for collaborating health professions and can therefore serve as a common goal.

Mentors remain important; however, everyday social interactions also impart a large influence on professional identity narratives. Participants’ stories consistently referenced friends, family, mentors, social relationships (e.g., neighbors), and social media as figures. Role models have been previously established as central characters in student IPC identity narratives (28). While mentors (i.e., other veterinarians) were influential in shaping a wide view of veterinary identity, other out-group influences seemed to hold similar power in their identity construction as reified by stories that seemed to take hold as master narratives from social media. These everyday social interactions were often interpreted through a shared lens, or shared social identity, in this sense, a shared social professional identity as shaped by social media (45). Participants would position these portrayals as a shared in-group reality, regardless of their personal experiences, to communicate their status within the veterinary identities. Veterinarians were situated as the figures portrayed on social media: a social identity combating perceived low status by educating non-veterinarians about veterinary skills and responsibilities. Estrada et al. (8) similarly found that veterinary students described a need to educate other professions and the public about the necessity of veterinary expertise and how it benefits society. More research is needed to better understand the complex interface of social media, shared social identity, and professional identities of veterinarians.

Social relationships with other professionals positively situated veterinary students toward IPC work while simultaneously situating them in a defensive posture to establish veterinary status. Participants described casual conversations with professionals in fields outside of veterinary medicine to form connections and engage in collaborative work. These conversations also highlighted differences in how veterinarians and other healthcare professionals work; however, these differences were deemed a space requiring understanding and growth. Participants also drew on stories from mentors outside of traditional veterinary practice to discuss the need and complications of interprofessional work. These social aspects impacted the curation of interprofessional identity more positively than for veterinarian identity. Rees et al. similarly concluded that a multiplicity of identities is constructed by clinicians and students within interprofessional interactions (33). Participant narratives provide context to explain previously identified differences in identity, group work, and role expectations among medical, veterinary, and MPH students (27, 28, 30). Successful interprofessional collaborations must acknowledge and navigate these tensions.

These narratives provided a deeper understanding of RIPLS variation through the discursive development of multiple professional identities in veterinary students. These findings suggest the RIPLS may not be appropriate “as is” for veterinary students, similar to other work (16, 18–22). In total, eight out of the nine participants expressed confusion regarding the RIPLS instrument and desired clarity to locate their identities within the presented constructs. RIPLS items within positive professional identity and roles and responsibilities were common areas of confusion. Participants grappled with similar words and phrases within challenging items. For example, one student explained that she would answer Item 15 (Shared learning will help to clarify the nature of patient problems) as neutral for the sole reason of item wording and issues with clarity. Interestingly, a suggested refinement of the RIPLS to a smaller set of items recommends utilizing this item (21), while our findings strongly suggest the wording troubles participants and skews responses. Due to the infancy of IPE/C studies in veterinary contexts, it is also difficult to determine whether the nature of participant confusion stems from confusion surrounding their identities, instrument failure, or a combination of these factors. These discussions highlight the importance of think-aloud interviews or similar qualitative strategies to bring context to participant survey choices and refine instruments (36, 37).

Multiple instruments are used across the field of research assessing interprofessional competencies, with one recent review finding the Interprofessional Education Collaborative scale (IPEC) as the most frequently used and the RIPLS as the second most used (46). The question of which tools and refinements of those tools are most reliable in assessing veterinary student identity and readiness for interprofessional collaboration is new and requires further research. Mixed methods approaches and qualitative focus group data have been used in recent work investigating other student groups (47, 48). Others have pointed out that more work is needed to determine the strengths of quantitative vs. qualitative data and reconcile those approaches (46). Our study strongly suggests that including qualitative methods provides vital information to inform these choices.

### 4.1 Limitations

This study is limited by the cross-sectional nature and drawing stories from a single site. Qualitative data limit the generalizability of findings, and more work is needed to explore these concepts across professional programs and after graduates enter practice. Although use of the RIPLS as a nidus for think aloud interviews may have necessarily limited the range of stories shared, this limitation was a methodologic choice. Although this tool serves an important role in IPE/IPC research and orientations of MD, DVM, and MPH students, it also has limitations that must be considered when using data to inform collaborative programs.

The RIPLS is a commonly utilized tool to determine the competencies of various professional student groups relevant to IPC (46). It is also the only tool in the literature to date selected to investigate veterinary student readiness for interprofessional learning (10–12). In an effort to build upon the small pool of existing work describing veterinary student identity and orientations to IPC, the RIPLS was a guide for think aloud interviews to qualitatively explore the veterinary student perspective and better describe highly intercorrelated constructs. Selecting the RIPLS facilitates conversation with previous work using the same tool and provides an opportunity to ponder previously identified limitations.

Other work troubles the RIPLS as a reliable instrument for interprofessional fields (49). Additionally, multiple quantitative studies using the RIPLS identify weak validity of constructs (16, 18–21). As a result of differing statistical analyses, researchers have also generated many versions of a refined RIPLS, and items or subscales refined are inconsistent across the literature. There are groups arguing for discontinued use of RIPLS altogether (16, 50) and others who suggest continued use with refinement (19–21). Determining the most appropriate tool for this study in veterinary contexts requires further research with larger sample sizes, ideally applying a mixed methods approach across institutions.

## 5 Conclusion

Veterinarian identities contain multitudes, are contradictory, and are often articulated as dual identities of veterinarian and veterinary interprofessional. One Health resonates strongly with veterinary identities and stands out as an important context for IPC, inclusive of veterinarians. Mentorship and social interactions in a variety of contexts remain important for identity development and warrant further work, with a particular consideration of social media. The RIPLS requires refinements and further validation as an instrument within veterinary medicine, given the One Health focus of the veterinary community.

## Data Availability

The datasets presented in this article are not readily available because IRB approval of this project included the provision that only the investigators would have access to the data. Requests to access the datasets should be directed to Tamara Hancock, HancockTS@missouri.edu.
